# Supplementary material for: The association between vitamin D status and COVID-19 in England: A cohort study using UK Biobank
Source: PLoS One. 2022 Jun 6;17(6):e0269064. doi: 10.1371/journal.pone.0269064 (PMC9170112; doi:10.1371/journal.pone.0269064)
Supplement: S1 Table — (DOCX) [file pone.0269064.s001.docx]

**S1 Table. The comparison of inclusion and exclusion participants**

|  | Included (N=307,512) | Excluded (N=194,977) |
| --- | --- | --- |
| Sex |  |  |
| - Female | 169,018 (55.0%) | 104,357 (53.5%) |
| - Male | 138,494 (45.0%) | 90,619 (46.5%) |
| Age^1^ |  |  |
| - Under 70 years old | 150,428 (48.9%) | 96,903 (49.7%) |
| - Greater and equal to 70 years old | 157,084 (51.1%) | 98,073 (50.3%) |
| Ethnicity |  |  |
| - White | 289,165 (94.0%) | 183,514 (94.1%) |
| - Black | 5,310 (1.7%) | 2,751 (1.4%) |
| - Asian and others | 13,037 (4.2%) | 8,712 (4.5%) |
| BMI^2^ |  |  |
| - Healthy weight | 1,480 (0.5%) | 1,146 (0.6%) |
| - Underweight | 97,499 (31.8%) | 62,790 (32.5%) |
| - Overweight | 130,370 (42.6%) | 81,870 (42.4%) |
| - Obese | 76,989 (25.1%) | 47,254 (24.5%) |
| Drinking frequency |  |  |
| - Never | 24,394 (8.0%) | 16,245 (8.4%) |
| - Sometimes | 70,806 (23.1%) | 43,056 (22.2%) |
| - Weekly | 149,866 (48.8%) | 94,857 (48.9%) |
| - Daily | 61,770 (20.1%) | 39,997 (20.6%) |
| Drinking status |  |  |
| - Never | 13,434 (4.4%) | 8,951 (4.6%) |
| - Previous | 10,867 (3.5%) | 7,235 (3.7%) |
| - Current | 282,442 (92.1%) | 177,910 (91.7%) |
| Smoking status |  |  |
| - Non-smoker | 167,513 (54.8%) | 106,004 (54.8%) |
| - Ex-smoker | 108,326 (35.4%) | 64,724 (33.4%) |
| - Current-smoker | 30,105 (9.8%) | 22,872 (11.8%) |
| IMD^3^ |  |  |
| - Least deprived | 59,870 (20.0%) | 38,261 (20.1%) |
| - 2 deprived | 59,219 (19.8%) | 38,773 (20.3%) |
| - 3 deprived | 60,261 (20.1%) | 37,570 (19.7%) |
| - 4 deprived | 60,255 (20.1%) | 37,634 (19.7%) |
| - Most deprived | 59,490 (19.9%) | 38,419 (20.2%) |
| Vitamin D and mineral supplementation^4^ |  |  |
| - Not taking supplement | 22417 (21.2%) | 14395 (22.8%) |
| - Taking vitamin D supplement | 83131 (78.8%) | 48692 (77.2%) |
| Vitamin D prescription |  |  |
| - Not receiving prescriptions | 234411 (76.2%) | 176220 (90.4%) |
| - Had vitamin D prescriptions | 73101 (23.8%) | 18757 (9.6%) |
| Regions |  |  |
| - East Midlands | 24,467 (8.0%) | 5,869 (4.1%) |
| - London | 44,374 (14.4%) | 16,917 (11.8%) |
| - North East | 43,707 (14.2%) | 8,570 (6.0%) |
| - North West | 50,808 (16.5%) | 19,454 (13.6%) |
| - South East | 28,859 (9.4%) | 10,955 (7.7%) |
| - South West | 29,445 (9.6%) | 9,424 (6.6%) |
| - West Midlands | 31,522 (10.3%) | 8,506 (5.9%) |
| - Yorkshire and The Humber | 54,330 (17.7%) | 11,768 (8.2%) |
| - Wales | 0 (0.0%) | 19,154 (13.4%) |
| - Scotland | 0 (0.0%) | 32,419 (22.7%) |
| Clinically vulnerable to COVID-19^5,6^ |  |  |
| - Not extremely vulnerable | 249,944 (81.3%) | 171,488 (88.0%) |
| - Clinically extremely vulnerable | 57,568 (18.7%) | 23,489 (12.0%) |
| Underlying comorbidities^5,7^ |  |  |
| - No chronic diseases | 94,237 (30.6%) | 107,046 (54.9%) |
| - Chronic diseases | 213,275 (69.4%) | 87,931 (45.1%) |

1. Calculated from participants' year of birth. 2. The classification is suggested by NICE guidelines. 3. IMD scores were classified by quintile. 4. Vitamin D supplement includes vitamin D, multivitamin, fish oil and calcium supplementation. 5. Health conditions were identified from linked electronic health records. 6. The clinically extremely vulnerable groups were defined by using Public Health England’s definition. 7. Including hypertension, cardiovascular diseases, diabetes mellitus, and asthma.
