# Supplementary material for: The association between vitamin D status and COVID-19 in England: A cohort study using UK Biobank
Source: PLoS One. 2022 Jun 6;17(6):e0269064. doi: 10.1371/journal.pone.0269064 (PMC9170112; doi:10.1371/journal.pone.0269064)
Supplement: S2 Table — (DOCX) [file pone.0269064.s002.docx]

**S2 Table. the association between receiving vitamin D tests during British summer time and serum vitamin D status**

|  | Vitamin D deficiency  (25OHD<25nmol/L) | | Vitamin D insufficiency (25OHD<50nmol/L) | |
| --- | --- | --- | --- | --- |
| British summer time^1^ | OR (crude) | OR (adjusted for all covariates^2^) | OR (crude) | OR (adjusted for all covariates^2^) |
| Non-summertime | - | - | - | - |
| British summer time | 0.34 (0.33-0.35, p<0.01) | 0.51 (0.58-0.75, p<0.01) | 0.4 (0.39-0.4, p<0.01) | 0.58 (0.57-0.59, p<0.01) |

1. British summer time: from April to October; non-summertime: from November to March; 2. Including sex, age, ethnicity, smoking, drinking frequency, index of multiple deprivation
