# Supplementary material for: The association between vitamin D status and COVID-19 in England: A cohort study using UK Biobank
Source: PLoS One. 2022 Jun 6;17(6):e0269064. doi: 10.1371/journal.pone.0269064 (PMC9170112; doi:10.1371/journal.pone.0269064)
Supplement: S7 Table — (DOCX) [file pone.0269064.s007.docx]

**S7 Table. The association between vitamin D supplementation and hospitalization due to Covid-19**

|  |  | HR (crude) | HR (adjusted for sex and age) | HR (adjusted for all covariates) |
| --- | --- | --- | --- | --- |
| **British summertime** (15 March to 25 October 2020) | No vitamin D supplementation | - | - | - |
|  | Taking vitamin D supplementation | 0.67 (0.53-0.84, p<0.01) | 0.71 (0.56-0.89, p=0.003) | 0.83 (0.66-1.06, p=0.139) |
| **Non-summertime** (26 October to 18 January 2021) | No vitamin D supplementation | - | - | - |
|  | Taking vitamin D supplementation | 0.86 (0.52-1.44, p=0.58) | 0.86 (0.52-1.44, p=0.574) | 0.83 (0.49-1.42, p=0.501) |
| Sex | Female |  | - | - |
|  | Male | 1.96 (1.76-2.18, p<0.01) | - | 1.72 (1.41-2.11, p<0.001) |
| Age^1^ | Under 70 years old | - | - | - |
|  | Greater and equal to 70 years old | 1.80 (1.61-2.01, p<0.01) | - | 1.29 (1.04-1.59, p=0.019) |
| Ethnicity | White | - | - | - |
|  | Black | 2.22 (1.67-2.95, p<0.01) | 2.75 (2.07-3.66, p<0.01) | 1.97 (1.23-3.15, p=0.005) |
|  | Asian and others | 1.59 (1.28-1.97, p<0.01) | 1.77 (1.43-2.20, p<0.01) | 1.34 (0.89-2.03, p=0.160) |
| BMI^2^ | Healthy weight | - | - | - |
|  | Underweight | 2.00 (0.94-4.23, p=0.07) | 2.31 (1.09-4.89, p=0.03) | 1.41 (0.35-5.75, p=0.633) |
|  | Overweight | 1.80 (1.55-2.10, p<0.01) | 1.56 (1.33-1.82, p<0.01) | 1.39 (1.06-1.83, p=0.017) |
|  | Obese | 3.05 (2.61-3.55, p<0.01) | 2.76 (2.37-3.22, p<0.01) | 1.93 (1.46-2.56, p<0.001) |
| Drinking frequency | Never | - | - | - |
|  | Sometimes | 0.67 (0.56-0.80, p<0.01) | 0.70 (0.58-0.83, p<0.01) | 0.85 (0.62-1.18, p=0.335) |
|  | Weekly | 0.53 (0.45-0.63, p<0.01) | 0.48 (0.41-0.57, p<0.01) | 0.68 (0.50-0.94, p=0.018) |
|  | Daily | 0.53 (0.43-0.64, p<0.01) | 0.43 (0.35-0.52, p<0.01) | 0.63 (0.44-0.92, p=0.016) |
| Smoking status | Non-smoker | - | - | - |
|  | Ex-smoker | 1.61 (1.44-1.81, p<0.01) | 1.41 (1.26-1.59, p<0.01) | 1.34 (1.08-1.67, p=0.007) |
|  | Current smoker | 1.94 (1.65-2.28, p<0.01) | 1.88 (1.60-2.22, p<0.01) | 1.40 (1.02-1.93, p=0.038) |
| Vitamin D status testing time | During non-summertime | - | - | - |
|  | During British summer time | 0.99 (0.89-1.11, p=0.91) | 1.01 (0.90-1.13, p=0.87) | 0.98 (0.80-1.20, p=0.876) |
| IMD^3^ | Least deprived | - | - | - |
|  | 2 deprived | 1.19 (0.96-1.46, p=0.11) | 1.19 (0.96-1.47,p=0.10) | 1.48 (1.01-2.18, p=0.044) |
|  | 3 deprived | 1.37 (1.12-1.68, p<0.01) | 1.39 (1.13-1.70, p<0.01) | 1.48 (1.01-2.16, p=0.045) |
|  | 4 deprived | 1.82 (1.50-2.20, p<0.01) | 1.88 (1.55-2.28, p<0.01) | 1.63 (1.12-2.36, p=0.011) |
|  | Most deprived | 2.87 (2.39-3.43, p<0.01) | 3.04 (2.54-3.64, p<0.01) | 1.97 (1.37-2.85, p<0.001) |
| Regions | North East | - | - | - |
|  | East Midlands | 1.18 (0.94-1.48, p=0.16) | 1.17 (0.93-1.47, p=0.191) | 1.60 (1.02-2.52, p=0.041) |
|  | London | 0.88 (0.71-1.09, p=0.24) | 0.92 (0.74-1.13, p=0.424) | 1.17 (0.79-1.74, p=0.438) |
|  | North West | 1.54 (1.28-1.84, p<0.01) | 1.52 (1.27-1.83, p<0.001) | 1.63 (1.14-2.33, p=0.008) |
|  | South East | 0.49 (0.37-0.66, p<0.01) | 0.49 (0.37-0.66, p<0.001) | 0.86 (0.50-1.49, p=0.595) |
|  | South West | 0.58 (0.44-0.76, p<0.01) | 0.60 (0.45-0.78, p<0.001) | 0.63 (0.35-1.13, p=0.120) |
|  | West Midlands | 1.34 (1.09-1.65, p<0.01) | 1.33 (1.08-1.64, p=0.007) | 1.53 (1.02-2.30, p=0.038) |
|  | Yorkshire and The Humber | 1.20 (1.00-1.45, p=0.05) | 1.21 (1.00-1.46, p=0.045) | 1.53 (1.05-2.22, p=0.026) |
| Clinically vulnerable to COVID-19^4^ | Not vulnerable | - | - | - |
|  | Extremely vulnerable | 3.50 (3.14-3.89, p<0.01) | 3.20 (2.87-3.57, p<0.001) | 2.75 (2.25-3.36, p<0.001) |
| Underlying comorbidities^5^ | No chronic diseases | - | - | - |
|  | Chronic diseases | 2.84 (2.43-3.31, p<0.01) | 2.41 (2.06-2.82, p<0.001) | 2.05 (1.50-2.80, p<0.001) |

1. Calculated from participants' year of birth. 2. The classification is suggested by NICE guidelines. 3. IMD scores were classified by quintile. 4. The clinically extremely vulnerable groups were defined by using Public Health England’s definition. 5. Including hypertension, cardiovascular diseases, diabetes mellitus, and asthma
