# Supplementary material for: The association between vitamin D status and COVID-19 in England: A cohort study using UK Biobank
Source: PLoS One. 2022 Jun 6;17(6):e0269064. doi: 10.1371/journal.pone.0269064 (PMC9170112; doi:10.1371/journal.pone.0269064)
Supplement: S12 Table — (DOCX) [file pone.0269064.s012.docx]

S12 Table. The data recording dates and data access dates

| **Dataset** | **Last recorded date** | **Access date** |
| --- | --- | --- |
| UK Biobank main dataset | 07 June 2013 | 14 May 2020 |
| COVID-19 laboratory testing results | 18 January 2021 | 29 January 2021 |
| Death registry | 18 December 2020 | 29 January 2021 |
| General practice clinical records | 25 July 2020 | 29 January 2021 |
| Hospitalization records | 30 November 2020 | 29 January 2021 |
