# Supplementary figures and images for: The association between vitamin D status and COVID-19 in England: A cohort study using UK Biobank
Source: PLoS One. 2022 Jun 6;17(6):e0269064. doi: 10.1371/journal.pone.0269064 (PMC9170112; doi:10.1371/journal.pone.0269064)

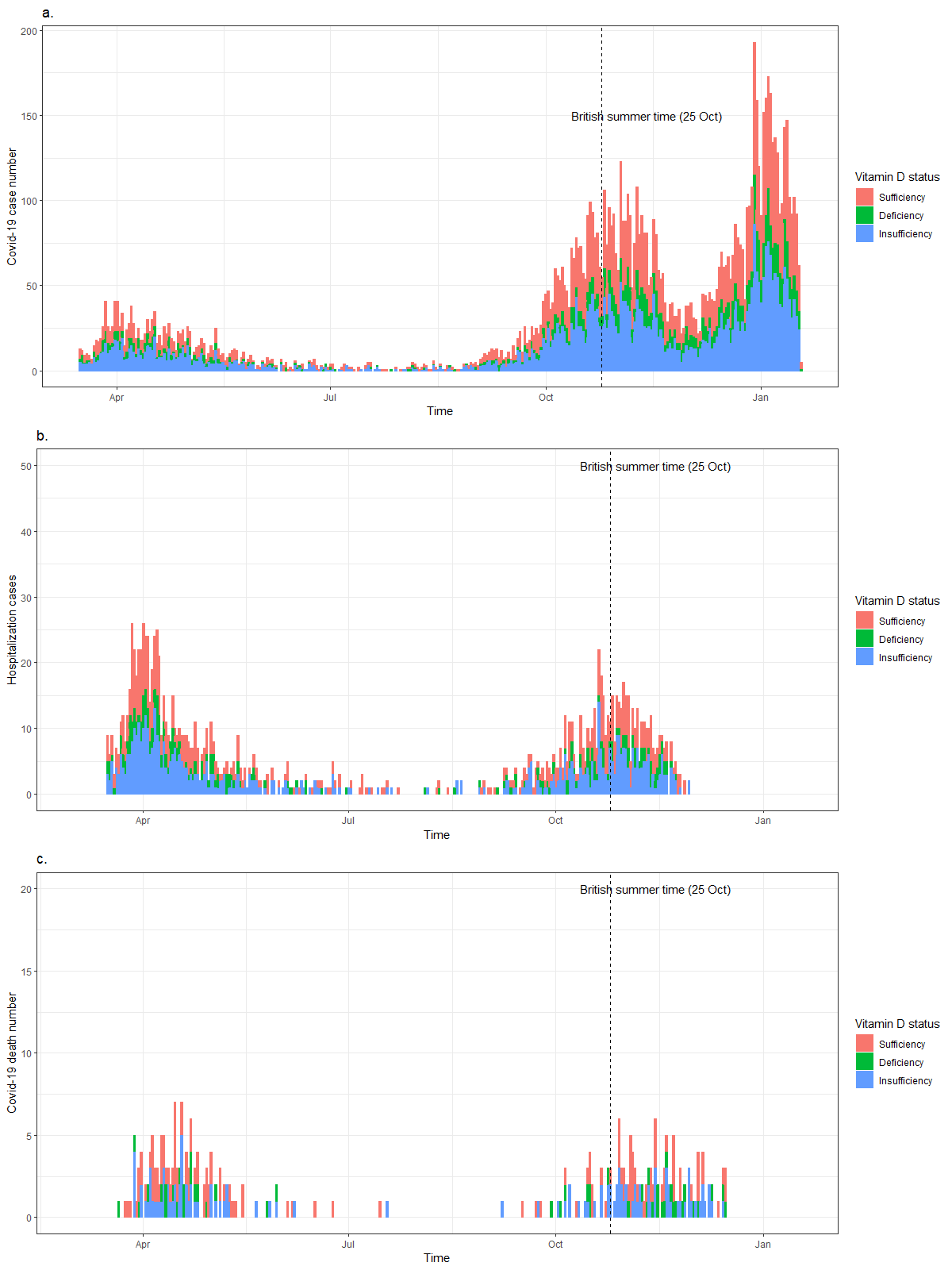


**S1 Figure. The distribution of COVID-19 outcomes.**

Supplement: S1 Fig — (DOCX) [file pone.0269064.s013.docx]
