# Supplementary material for: The association between vitamin D status and COVID-19 in England: A cohort study using UK Biobank
Source: PLoS One. 2022 Jun 6;17(6):e0269064. doi: 10.1371/journal.pone.0269064 (PMC9170112; doi:10.1371/journal.pone.0269064)
Supplement: S2 Fig — (DOCX) [file pone.0269064.s014.docx]

**S2 Figure. The log(-log(survival)) plot of Cox models for the primary and secondary outcomes**
