# Supplementary material for: The association between vitamin D status and COVID-19 in England: A cohort study using UK Biobank
Source: PLoS One. 2022 Jun 6;17(6):e0269064. doi: 10.1371/journal.pone.0269064 (PMC9170112; doi:10.1371/journal.pone.0269064)
Supplement: Attachment — Submitted filename: Response to Reviewers.docx [file pone.0269064.s015.docx]

**Journal requirements:**

When submitting your revision, we need you to address these additional requirements.

1. Please ensure that your manuscript meets PLOS ONE's style requirements, including those for file naming. The PLOS ONE style templates can be found at

[https://journals.plos.org/plosone/s/file?id=wjVg/PLOSOne_formatting_sample_main_body.pdf](https://protect-eu.mimecast.com/s/cisxCQ7METooVNYhMn47S?domain=journals.plos.org)  and

[https://journals.plos.org/plosone/s/file?id=ba62/PLOSOne_formatting_sample_title_authors_affiliations.pdf](https://protect-eu.mimecast.com/s/ARyxCR1NGc55YgxcOVykp?domain=journals.plos.org)

2. Thank you for providing the date(s) when patient medical information was initially recorded. Please also include the date(s) on which your research team accessed the databases/records to obtain the retrospective data used in your study.

We have summarized the last recorded date and our data access dates as the following table, which has been included as **S12 Table**.

S12 Table. The data recording dates and data access dates

| **Dataset** | **Last recorded date** | **Access date** |
| --- | --- | --- |
| UK Biobank main dataset | 07 June 2013 | 14 May 2020 |
| COVID-19 laboratory testing results | 18 January 2021 | 29 January 2021 |
| Death registry | 18 December 2020 | 29 January 2021 |
| General practice clinical records | 25 July 2020 | 29 January 2021 |
| Hospitalization records | 30 November 2020 | 29 January 2021 |

3. In your ethics statement in the Methods section and in the online submission form, please provide additional information about the data used in your retrospective study. Specifically, please ensure that you have discussed whether all patient data were fully anonymized before you accessed them and/or whether the IRB or ethics committee waived the requirement for informed consent. If patients provided informed written consent to have data from their medical records used in research, please include this information.

We have revised the ethic statement as follows (p13, line 217-228):

The UK Biobank data had been de-identified by removing personal data fields, such as postcodes and dates of birth [1]. Finally, the de-identified and anonymous data were released to eligible researchers for study purposes only. UK Biobank already has its Research Tissue Bank (RTB) approval from its Research Ethics Committee (REC), which covers most usage of the data under the UK Biobank ethics and governance framework [2]. The UK Biobank project was also approved by the Northwest Haydock Research Ethics Committee (reference: 11/NW/0382). Our project was approved by UK Biobank (ID:51265) and by the Research Ethics Committee of the London School of Hygiene and Tropical Medicine (reference: 17158). For COVID-19 data, an approved UK Biobank project will be automatically authorised to conduct COVID-19 related research after registering to access COVID-19 data [3]. We followed the principles of the Declaration of Helsinki[4].

Regarding the patient consent, we revised the section first paragraph in the method section by citing the supporting references and documents as follows (p.7, line 93-95):

… UK Biobank participants also gave their consent to have electronic health records linked, including primary care and inpatient care records, and death certificates [5, 6].

4. Please include your actual numerical p-values in Tables 3-5 and supplementary tables 2-11

Thank you for the comment. We have now added the p-value to Tables 3-5 and S2-11 Tables.

5.  Thank you for stating the following in the Funding Section of your manuscript:

“LYL is funded by the scholarship of government sponsorship for overseas study by the Ministry of Education Republic of China (Taiwan). RM is funded by a Sir Henry Wellcome Postdoctoral Fellowship (201375/Z/16/Z). CWG is supported by a Wellcome Intermediate Clinical Fellowship (201440/Z/16/Z). SML is funded by a Wellcome Senior Clinical Fellowship in Science (205039/Z/16/Z).”

We note that you have provided additional information within the Funding Section that is not currently declared in your Funding Statement. Please note that funding information should not appear in other areas of your manuscript. We will only publish funding information present in the Funding Statement section of the online submission form.

Please remove any funding-related text from the manuscript and let us know how you would like to update your Funding Statement. Currently, your Funding Statement reads as follows:

 “The authors received no specific funding for this work.”

Please include your amended statements within your cover letter; we will change the online submission form on your behalf.

We would like to replace the funding statement using the following text (p1, line 14-19):

“LYL is funded by the scholarship of government sponsorship for overseas study by the Ministry of Education Republic of China (Taiwan). RM is funded by a Sir Henry Wellcome Postdoctoral Fellowship (201375/Z/16/Z). CWG is supported by a Wellcome Intermediate Clinical Fellowship (201440/Z/16/Z). SML is funded by a Wellcome Senior Clinical Fellowship in Science (205039/Z/16/Z).”

In addition, we have now added this paragraph to the cover letter.

6. In your Data Availability statement, you have not specified where the minimal data set underlying the results described in your manuscript can be found. PLOS defines a study's minimal data set as the underlying data used to reach the conclusions drawn in the manuscript and any additional data required to replicate the reported study findings in their entirety. All PLOS journals require that the minimal data set be made fully available. For more information about our data policy, please see [http://journals.plos.org/plosone/s/data-availability](http://protect-eu.mimecast.com/s/yzSOCVQRMUggBPYuQaoZy?domain=journals.plos.org).

Upon re-submitting your revised manuscript, please upload your study’s minimal underlying data set as either Supporting Information files or to a stable, public repository and include the relevant URLs, DOIs, or accession numbers within your revised cover letter. For a list of acceptable repositories, please see [http://journals.plos.org/plosone/s/data-availability#loc-recommended-repositories](http://protect-eu.mimecast.com/s/GQcsCWqVNfDD3yVIpwT9b?domain=journals.plos.org). Any potentially identifying patient information must be fully anonymized.

Important: If there are ethical or legal restrictions to sharing your data publicly, please explain these restrictions in detail. Please see our guidelines for more information on what we consider unacceptable restrictions to publicly sharing data: [http://journals.plos.org/plosone/s/data-availability#loc-unacceptable-data-access-restrictions](http://protect-eu.mimecast.com/s/8H_hCX5WOIOOAMWu7-Yh2?domain=journals.plos.org). Note that it is not acceptable for the authors to be the sole named individuals responsible for ensuring data access.

We will update your Data Availability statement to reflect the information you provide in your cover letter.

We have revised the data availability statement as follows (p2, line 21-29):

The database access dates were summarized in supporting information (**S12 Table**). The analytic codes of this project are shared on GitHub for review and re-use under MIT open license (<https://github.com/liang-yu12/vd_covid>). Relevant data information about the project can be accessed using the reference number "UK Biobank Application 51265." Complete pseudonymised participant data is not openly shared under the material transfer agreement with UK Biobank and ethics approval. Other eligible researchers can apply for UK Biobank data to answer specific research questions. Further information about applying for data access can be obtained from the UK Biobank website (<https://www.ukbiobank.ac.uk>) or by emailing UK Biobank ([ukbiobank@ukbiobank.ac.uk](mailto:ukbiobank@ukbiobank.ac.uk)).

7. Please include captions for your Supporting Information files at the end of your manuscript, and update any in-text citations to match accordingly. Please see our Supporting Information guidelines for more information: [http://journals.plos.org/plosone/s/supporting-information](http://protect-eu.mimecast.com/s/4uFHCY58PIAAY6wcjobBH?domain=journals.plos.org).

Thank you. We have now included the supporting information at the end of our manuscript (p50, line 634–656).

**Response to reviewer**

Reviewer #1: This could be an important paper that is worth of publication but has multiple major flaws that must be addressed before it can be seriously considered for publication,

We thank the reviewer for the helpful comments. We noticed some coding errors in our analysis, which previously over diagnosed people who were extremely vulnerable to COVID-19. These errors now have been corrected as follows (page 16, line 257):

**Table 2. The distribution of demographic characteristics by vitamin D status**

|  | Total (N=307,512) | Vitamin D deficiency  (<25 nmol/L)  (N=37,763) | Vitamin D insufficiency (25 – 50 nmol/L) (N=126,802) | Vitamin D sufficiency (≥ 50 nmol/L) (N=142,947) |  |
| --- | --- | --- | --- | --- | --- |
| Clinically vulnerable to COVID-19^6,7^ |  |  |  |  |  |
| - Not vulnerable | 249,944 (81.3%) | 29,903 (79.2%) | 103,063 (81.3%) | 116,978 (81.8%) |  |
| - Clinically extremely vulnerable | 57,568 (18.7%) | 7,860 (20.8%) | 23,739 (18.7%) | 25,969 (18.2%) |  |

6. Health conditions were identified from linked electronic health records. 7. The clinically extremely vulnerable groups were defined by using Public Health England’s definition.

We have addressed other comments as below:

1. The authors are unclear about the primary outcome, which I think is a diagnosis of COVID 19.

Thank you for the comment. The reviewer is correct. We have revised the words in the methods sections as follows to clarify (page 8, line 116–128):

**Primary outcome: diagnosis of COVID-19**

The primary outcome of our study was the diagnosis of COVID-19, which was defined through laboratory testing or by clinical diagnosis of COVID-19. The laboratory tests for SARS-CoV-2 infection were performed using PCR, which was performed by the NHS (Pillar 1) or commercial partners (Pillar 2) (18, 19). These testing results were reported to Public Health England and automatically imported into UK Biobank weekly (20). Clinically diagnosed COVID-19 was defined as participants diagnosed with COVID-19 codes in their electronic health records, either in primary care or inpatient care, or on the death certificate. We used existing code lists in CTV3 codes, SNOMED-CT and ICD-10 to identify the diagnosis of COVID-19.

2. There is insufficient distinction made between " not statistically significant" and underpowered for the outcome. The hospitalization and mortality outcomes are underpowered for likely and clinically important effect sizes

Thank you for this comment. We agree with the referee that the wide confidence intervals and large p-values for the hospitalization and mortality outcomes indicate that our results were imprecise, which implies they were underpowered to detect the effects we presented [7]. With our large SEs, we may have been able to detect larger hospitalization/mortality effects if they existed in our data, but they did not. We have clarified in the text that the confidence intervals were consistent with other clinically important effects in increasing the risk of COVID-19 as well as no effect, none of which can be ruled out by our analysis. We have address this in the discussion (page 39, line 392–397):

“… Finally, our analysis showed that some known factors were also associated with COVID-19 hospitalisation and mortality, including male sex, older age, non-white ethnicity, abnormal BMI, cigarette smoking, being more deprived, being clinically vulnerable and having underlying comorbidities. These findings were similar to previous studies using a large electronic health records database [32], implying that our analysis regarding hospitalisation and mortality is valid.”

3. There is some attention given to the historical nature of the vitamin D levels but the import of those lags and likely interval treatments and changes is not seriously enough appreciated in the conclusions of the paper nor are there any efforts to discuss whether they can be usefully analyzed with this data.

The referee is right to point out the importance of discussing the effects of using historical vitamin D measurement. We have now addressed this point in the discussion as follows (page 40, line 400–407):

“A previous study among postmenopausal women repeatedly measured vitamin D levels after five years, and the results showed the intraclass correlation coefficient between the two results was only 0.59 (0.54–0.64), which was suboptimal [8]. Another study examined 15,473 people with repeated vitamin D level tests in UK Biobank data, which showed an 84% concordance rate after 4.3 years [9]. However, in our study, the vitamin D levels were measured seven to 15 years ago, which introduces a possible misclassification bias of exposure. The misclassification is likely to be non-differential, which could attenuate our estimates toward the null.”

In addition, we have also now addressed this limitation in a revised conclusion as follows (page 42, line 462–474):

“Our study shows inconsistent associations between vitamin D status and diagnosis of COVID-19, as well as no association between historical vitamin D status and the risk of hospital admission and mortality due to COVID-19. However, these results were limited by the potential for misclassification bias caused by the historical vitamin D status and the changing COVID-19 testing strategies. To precisely investigate the possible role of vitamin D in COVID-19 prevention, more studies using recent vitamin D status data and systematic COVID-19 surveillance will be needed. In addition, the results of the ongoing trial may provide more compelling evidence on the effects of vitamin D supplementation on preventing COVID-19 [10]. Because of the uncertainty of the association between vitamin D deficiency and the risk of COVID-19, there is currently insufficient evidence to support prioritizing vitamin D supplementation or fortification over other preventive strategies for COVID-19 such as mass vaccination programmes.”

4. The finding of significant association of vitamin D deficiency with covid-19 positivity controlling for covariates in the winter seems to be a major finding of the paper and is neglected by the authors. This is especially important because there are strong reasons to believe that summer sun exposure confounds vitamin D levels for COVID risk in the summer

Although we agree with the referee that there was some evidence of the association between vitamin D deficiency and COVID-19 during non-summertime, we would like to address the significant heterogeneity between follow-up periods. First, the stratification of follow-up period not only showed the potential difference in the sun exposure, but also reflected different properties of COVID-19 pandemic, such as different waves of the outbreak caused by more virulent variant strains, or different strategies for COVID-19 response. Because of these heterogeneity between two follow-up times, we would like to not over focus on a marginal significant result during non-summertime. Second, however, as mentioned in the previous paragraphs, the misclassification bias caused by the historical vitamin D levels and the testing strategies should also be considered. Because of these limitations, we cautiously interpreted our results as an inconsistent association between vitamin D status and diagnosis of COVID-19. We addressed these two points in the discussion as follows (page 41, line 444–451):

“However, the heterogeneity among the follow-up periods influences the association between vitamin D status and the diagnosis of COVID-19. During British summertime and non-summertime, there were two waves of COVID-19 pandemic caused by different circulating strains [11], and the government’s response to COVID-19 also varied. In addition, as previously discussed, the potential misclassification of vitamin D exposure and COVID-19 outcome also could have introduced biases, which may lead to inaccurate estimation in the association between vitamin D and COVID-19.“

5. I do not understand why results unadjusted for known risk factors such as gender are presented. This seems to muddy the presentation

We understand the reviewer’s concern. However, as can be seen in our tables, we considered that presenting crude, minimally adjusted, and fully adjusted estimates aids interpretation because it demonstrates both the extent of confounding, and the covariates responsible. Presenting all potential confounders in models is useful to demonstrate the effect of confounding.

6. The term non-British summertime is awkward. It sounds like summertime not in Britain.

We agree with the referee that using "non-British summertime" is misleading. Therefore, we've changed the term into "non-summertime" in the main text and figures.

7. The conclusion is disappointing in suggesting that this work suggests that vitamin D should not be prioritized over vaccination. That is clear and NOT the question. The question is there evidence vitamin D might play a protective role in COVID-19, and might it be worth studying or trying even outside of studies given the low risks. This is what the authors should be commenting on.

We agree with the referee that the study's aim, implications, and conclusion should be clearer. Therefore, we have revised our introduction and conclusion. We've revised the last paragraph of our introduction as follows (page 5, line 86–91):

“Despite this potential, the association between vitamin D and COVID-19 is still unclear. If vitamin D deficiency is associated with COVID-19, vitamin D supplementation may be a potential public health intervention. Consequently, we aimed to conduct a historical cohort study using UK Biobank dataset and linked electronic health records to better understand the association between serum vitamin D status, vitamin D supplementation and diagnosis of COVID-19 and outcomes.”

We also revised the conclusion as follows (page 42, line 462–474):

“Our study shows inconsistent associations between vitamin D status and diagnosis of COVID-19, as well as no association between historical vitamin D status and the risk of hospital admission and mortality due to COVID-19. However, these results were limited by the potential for misclassification bias caused by the historical vitamin D status and the changing COVID-19 testing strategies. To precisely investigate the possible role of vitamin D in COVID-19 prevention, more studies using recent vitamin D status data and systematic COVID-19 surveillance will be needed. In addition, the results of an ongoing trial may provide more compelling evidence on the effects of vitamin D supplementation on preventing COVID-19 [10]. Because of the uncertainty of the association between vitamin D deficiency and the risk of COVID-19, there is currently insufficient evidence to support prioritizing vitamin D supplementation or fortification over other preventive strategies for COVID-19 such as mass vaccination programmes.”

**References**

1.	UK Biobank. Summary de-identification protocol 2021 [updated 6 July 2021; cited 2021 4 December]. 2:[Available from: <https://www.ukbiobank.ac.uk/media/5bvp0vqw/de-identification-protocol.pdf>.

2.	UK Biobank. UK Biobank ethics and governance framework 2007 [updated October 2007; cited 2022 28 Feb]. 3.0:[Available from: <https://www.ukbiobank.ac.uk/media/0xsbmfmw/egf.pdf>.

3.	UK Biobank. UK Biobank - COVID-19 Data Release FAQs 2020 [cited 2020 26, May, 2020]. Available from: <https://www.ukbiobank.ac.uk/wp-content/uploads/2020/05/ACCESS_066V1.1.A.COVID-19-FAQs_v1.1_DRAFT.docxAMENDEDBH.pdf>.

4.	World Medical A. World Medical Association Declaration of Helsinki: ethical principles for medical research involving human subjects. JAMA. 2013;310(20):2191-4. Epub 2013/10/22. doi: 10.1001/jama.2013.281053. PubMed PMID: 24141714.

5.	Sudlow C, Gallacher J, Allen N, Beral V, Burton P, Danesh J, et al. UK biobank: an open access resource for identifying the causes of a wide range of complex diseases of middle and old age. PLoS Med. 2015;12(3):e1001779. Epub 2015/04/01. doi: 10.1371/journal.pmed.1001779. PubMed PMID: 25826379; PubMed Central PMCID: PMCPMC4380465.

6.	UK Biobank. Consent Form: UK Biobank.

7.	Althouse AD. Post Hoc Power: Not Empowering, Just Misleading. J Surg Res. 2021;259:A3-a6. Epub 20200816. doi: 10.1016/j.jss.2019.10.049. PubMed PMID: 32814615.

8.	Meng JE, Hovey KM, Wactawski-Wende J, Andrews CA, LaMonte MJ, Horst RL, et al. Intraindividual Variation in Plasma 25-Hydroxyvitamin D Measures 5 Years Apart among Postmenopausal Women. Cancer Epidemiology Biomarkers &amp;amp; Prevention. 2012;21(6):916. doi: 10.1158/1055-9965.EPI-12-0026.

9.	Hastie CE, Pell JP, Sattar N. Vitamin D and COVID-19 infection and mortality in UK Biobank. Eur J Nutr. 2021;60(1):545-8. Epub 2020/08/26. doi: 10.1007/s00394-020-02372-4. PubMed PMID: 32851419.

10.	Trial of vitamin D to reduce risk and severity of COVID-19 and other acute respiratory infections. Identifier: NCT04579640. 2020.

11.	Office for National Statistics. Coronavirus (COVID-19) Infection Survey technical article: waves and lags of COVID-19 in England, June 2021 2021 [updated 29 June 2021; cited 2022 4 March]. Available from: <https://www.ons.gov.uk/peoplepopulationandcommunity/healthandsocialcare/conditionsanddiseases/articles/coronaviruscovid19infectionsurveytechnicalarticle/wavesandlagsofcovid19inenglandjune2021>.
